# Acute Tetraplegia Caused by Rat Bite Fever in Snake Keeper and Transmission of *Streptobacillus moniliformis*

**DOI:** 10.3201/eid2304.161987

**Published:** 2017-04

**Authors:** Tobias Eisenberg, Simon Poignant, Youenn Jouan, Ahmad Fawzy, Werner Nicklas, Christa Ewers, Laurent Mereghetti, Antoine Guillon

**Affiliations:** Hessian State Laboratory, Giessen, Germany (T. Eisenberg, A. Fawzy);; Centre Hospitalier Universitaire de Tours, Tours, France (S. Poignant, Y. Jouan, L. Mereghetti, A. Guillon);; Université François Rabelais, Tours (S. Poignant, Y. Jouan, L. Mereghetti, A. Guillon);; Cairo University, Giza, Egypt (A. Fawzy);; Justus-Liebig-University, Giessen (A. Fawzy, C. Ewers);; German Cancer Research Center, Heidelberg, Germany (W. Nicklas)

**Keywords:** acute tetraplegia, rat bite fever, snake keeper, transmission, Streptobacillus moniliformis, bacteria, zoonoses, variable number tandem repeat analysis, multilocus variant analysis, snakes, reptiles, rats, PCR, species specificity, France

## Abstract

We report acute tetraplegia caused by rat bite fever in a 59-year old man (snake keeper) and transmission of *Streptobacillus moniliformis*. We found an identical characteristic bacterial pattern in rat and human samples, which validated genotyping-based evidence for infection with the same strain, and identified diagnostic difficulties concerning infection with this microorganism.

Human infections by *Streptobacillus moniliformis* are assumed to be caused by rats on the basis of epidemiologic information. We provide genotyping-based evidence for infection with the same bacterial strain in rat and human samples and highlight diagnostic difficulties concerning this microorganism and its potential for life-threatening consequences.

A 59-year-old man was admitted to Centre Hospitalier Universitaire de Tours (Tours, France) because he was unable to stand and had acute progressive onset of dyspnea and a 15-day history of fever and arthralgia (left knee, right wrist) but no signs of rash. He was sedated, mechanically ventilated, and admitted to the intensive care unit. The patient had a temperature of 39°C, a pulse rate of 63 beats/min, and a blood pressure of 126/68 mm Hg.

After discontinuation of sedation, physical examination showed cervical pain, flaccid tetraplegia, and sensitivity at the T4 level. His knees and left wrist were swollen and had joint effusions. There was little available information for the patient because he could not speak and had no known social contacts. Blood tests showed an increased leukocyte count (15 × 10^9^ cells/L), predominantly neutrophils, and an increased C-reactive protein level (125 mg/L). 

The patient was given antimicrobial drugs (amoxicillin and cloxacillin) after blood and synovia (knee) sampling. Cervical magnetic resonance imaging showed C5–T1 vertebral osteomyelitis and an epidural abscess with consecutive compression of the spinal cord (C5–T1) (Figure). Surgical spinal decompression and vertebral stabilization were not attempted because of extensiveness of injury and flaccid tetraplegia. Transthoracic and transesophageal echocardiograms showed no features of endocarditis. Blood cultures showed negative results. Joint effusions contained a culture-negative inflammatory liquid and uric acid crystals. The patient was given a tracheotomy and continuously ventilated.

**Figure:**
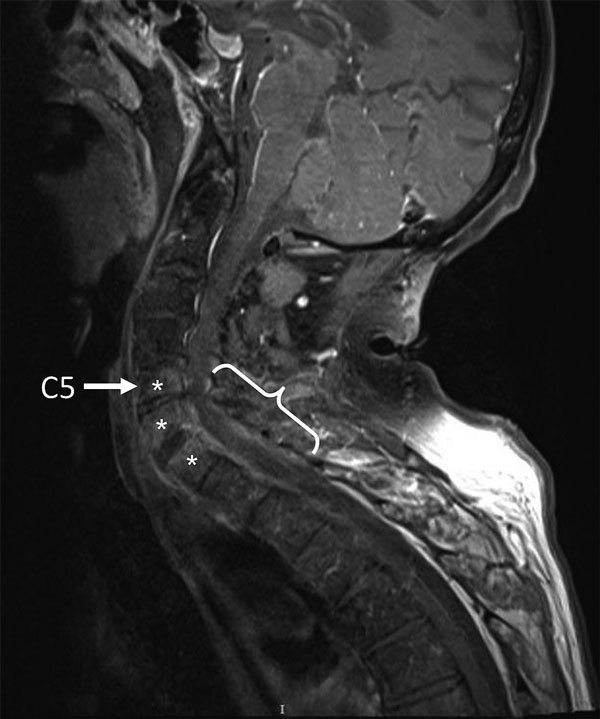
Fat-saturated, contrast-enhanced T1-weighted magnetic resonance image of the spine of a 59-year-old man (snake keeper) with rat bite fever. Sagittal view of the cervical spine shows spondylodiscitis (*) and an epidural absess with C5–T1 compression (brace). Preexisting spinal degeneration was observed and was probably a promoting factor for spinal compression.

A final diagnosis was obtained by sequencing the 16S rRNA gene obtained directly from synovia. An 897-nt partial 16S rRNA sequence showed 99.0% identity with sequences of *S. moniliformis* (GenBank accession nos. JQ087393 and CP001779).

The patient was a snake keeper who bred rats for snake food. He reported snake bites but not rat bites. We sampled his snakes (*Boa constrictor* and *Elaphe* sp.) and 1 of his feeder rats (*Rattus norvegicus*) by obtaining swab and biopsy specimens from oral cavities of all animals. All cultures were polymicrobial. We used desorption/ionization time-of-flight mass spectrometry (Bruker Daltonique, Wissembourg, France) to identify isolated bacteria but failed to identify *S. moniliformis*.

Synovia and serum samples from the patient and oral swab and biopsy specimens from animals were subjected to three 16S rRNA gene–based PCRs that were genus specific, rather than *S. moniliformis* specific (*1*). Synovia from the patient and 2 swab and 2 biopsy specimens from the same rat, but none of the oral samples from snakes, were positive. This result suggested rat bite fever. However, diagnosis of rat bite fever on the basis of partial 16S rRNA gene sequencing might be uncertain (*1*).

We tested the same samples by using *S. moniliformis*–specific multilocus variant analysis (MLVA) (*2*) to identify the bacterial transmission chain. Results were consistent with those for PCR and identified 2 MLVA genotypes of *S. moniliformis* in rat oral samples. Conversely, genomic information obtained for human synovia showed only 1 of these patterns, indicating a clonal relationship with 1 of the rat bacterial strains. Serum from the patient obtained on day 1 after hospitalization contained antibodies against *S. moniliformis* when tested by bead-based multiplex serologic analysis and indirect immunofluorescence.

Rat bite fever is an underdiagnosed worldwide zoonosis closely associated with bites of rats or close contact with them. Snakes that eat rats might serve, at least temporarily, as reservoirs for human infection. Thus, we attempted to detect the rat bite fever organism in oral and biopsy specimens from the patient’s snakes by using different PCRs (including a quantitative PCR that has an analytical sensitivity of 10 DNA molecules). All results were negative.

Four studies reported rat bite fever associated with keeping reptiles, but definitive transmission could not be proven in these instances, in which infections seemed more likely to be introduced by feeder rats (*3*–*6*). Regular contact with prey rats might be a general risk factor, and being bitten by a snake shortly after it fed on a prey rat might have the same consequences as a rat bite. In our study, we identified and typed the involved clone by using a recently developed, species-specific, culture-independent MLVA scheme (http://microbesgenotyping.i2bc.paris-saclay.fr/databases/public).

We showed that a rat might be simultaneously colonized by >1 clone of *S. moniliformis* and demonstrated identical strains in the human patient and the reservoir host (Technical Appendix Figure). The infectious genotype has been designated as LHL18 on the basis of the novel allele combination 17-3-16.

We demonstrated the presence of an identical characteristic *S. moniliformis* bacterial pattern in rat and human samples, which validated genotyping-based evidence for infection with the same strain. Our case highlights diagnostic difficulties concerning this microorganism and supports tropism of this bacteria for synovial tissue and its potential for life-threatening consequences.

Technical Appendix Additional information on acute tetraplegia caused by rat bite fever in snake keeper and transmission of *Streptobacillus moniliformis.*
