# Supplementary material for: Acute Tetraplegia Caused by Rat Bite Fever in Snake Keeper and Transmission of Streptobacillus moniliformis
Source: Emerg Infect Dis. 2017 Apr;23(4):719–21. doi: 10.3201/eid2304.161987 (PMC5367424; doi:10.3201/eid2304.161987)
Supplement: Technical Appendix — Additional information on acute tetraplegia caused by rat bite fever in snake keeper and transmission of Streptobacillus moniliformis. [file 16-1987-Techapp-s1.pdf]

Article DOI: http://dx.doi.org/10.3201/eid2304.161987

Acute Tetraplegia Caused by Rat Bite Fever in Snake Keeper and Transmission of *Streptobacillus moniliformis*

Technical Appendix

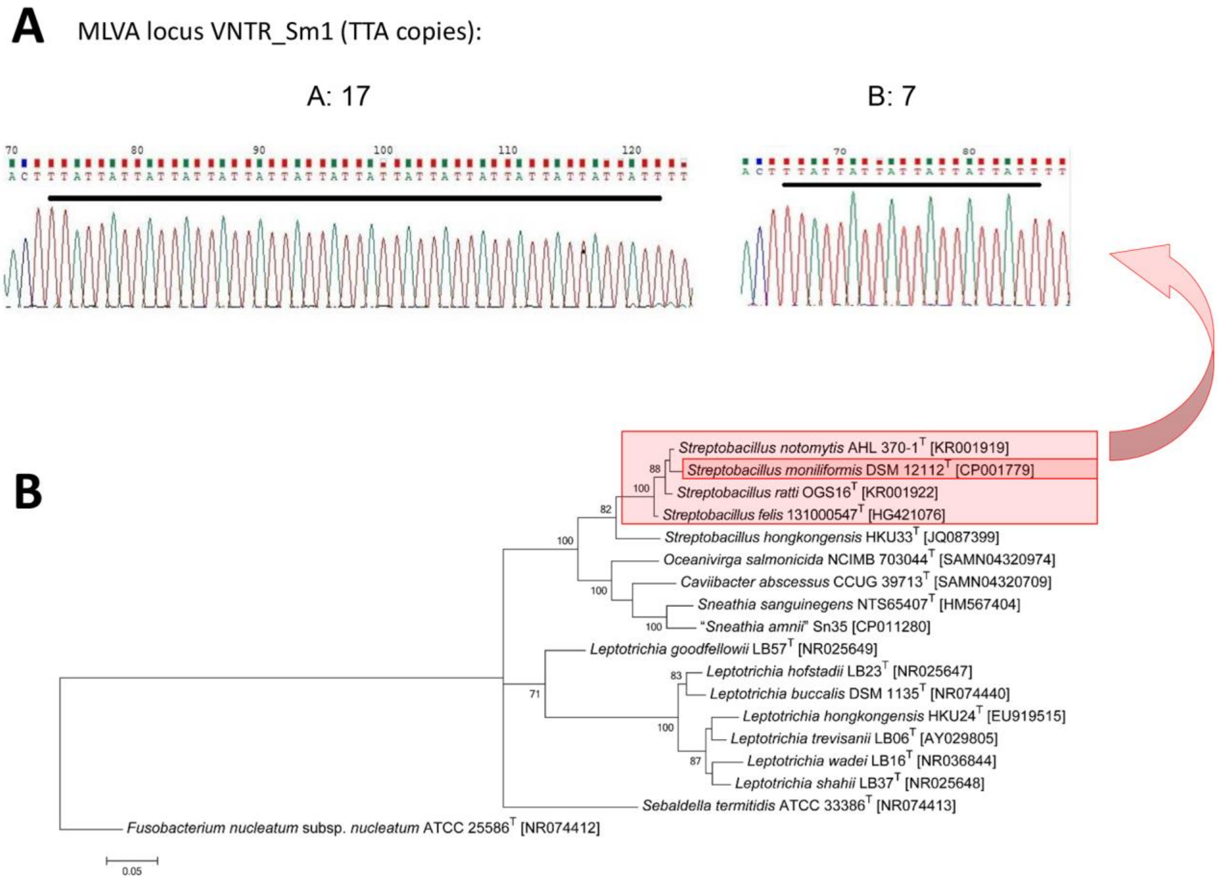

Technical Appendix Figure. A) *Streptobacillus moniliformis*–specific multilocus variant analysis (MLVA) for a 59-year-old man (snake keeper) with acute tetraplegia caused by rat bite fever. Chromatograms show sequencing results for variable number tandem repeat (VNTR) Sm1 locus amplified from 2 different rat oral samples. A: 16.7 (17) copies of the 3-nt repeat (TTA) (identical to the locus amplified from the human sample). B: 6.7 (7) copies of the same repeat. B) Maximum-likelihood tree showing phylogenetic position of *S. moniliformis* in the family *Leptotrichiaceae* on the basis of 16S rRNA gene sequences. Closely related species (>98% sequence homology) are shown in the red box. The tree was generated by using MEGA version 5.2.2 (http://www.megasoftware.net/) (Tamura-Nei model, gamma distribution plus invariant sites) and is based on 1,572 nt. GenBank accession numbers are indicated in brackets. Numbers at branch nodes indicate bootstrap values >70% (100 replicates).

Scale bar indicates nucleotide substitutions per site. *Fusobacterium nucleatum* subsp. *nucleatum* ATCC 25586^{T} was used as the outgroup.
